# The Complex Immunological Alterations in Patients with Type 2 Diabetes Mellitus on Hemodialysis

**DOI:** 10.3390/jcm13133687

**Published:** 2024-06-25

**Authors:** Maria-Florina Trandafir, Octavian Ionel Savu, Mihaela Gheorghiu

**Affiliations:** 1 Pathophysiology and Immunology Department, “Carol Davila” University of Medicine and Pharmacy, 020021 Bucharest, Romania; mihaela.gheorghiu@umfcd.ro; 2 Doctoral School, “Carol Davila” University of Medicine and Pharmacy, 020021 Bucharest, Romania; octavian.savu@umfcd.ro; 3 “N. C. Paulescu” National Institute of Diabetes, Nutrition and Metabolic Diseases, 020475 Bucharest, Romania

**Keywords:** diabetes mellitus, chronic hemodialysis, chronic inflammation, innate immunity, acquired immunity

## Abstract

It is widely known that diabetes mellitus negatively impacts both the innate immunity (the inflammatory response) and the acquired immunity (the humoral and cellular immune responses). Many patients with diabetes go on to develop chronic kidney disease, which will necessitate hemodialysis. In turn, long-term chronic hemodialysis generates an additional chronic inflammatory response and impairs acquired immunity. The purpose of this paper is to outline and compare the mechanisms that are the basis of the constant aggression towards self-components that affects patients with diabetes on hemodialysis, in order to find possible new therapeutic ways to improve the functionality of the immune system. Our study will take a detailed look at the mechanisms of endothelial alteration in diabetes and hemodialysis, at the mechanisms of inflammatory generation and signaling at different levels and also at the mechanisms of inflammation-induced insulin resistance. It will also discuss the alterations in leukocyte chemotaxis, antigen recognition and the dysfunctionalities in neutrophils and macrophages. Regarding acquired immunity, we will outline the behavioral alterations of T and B lymphocytes induced by diabetes mellitus and chronic hemodialysis.

## 1. Introduction

Diabetes mellitus includes the elevation of the serum glucose concentration, associated with a deficiency of glucose intake in insulin-dependent tissues, which causes secondary structural and functional changes in these tissues, through a chronic ATP deficiency. This condition generates multiple other diseases caused by macrovascular and microvascular damage that have the maximal negative impact on the brain, kidney, heart and eyes [1].

It has long been known that the presence of diabetes mellitus negatively influences both innate and acquired immunity, so much so that patients with diabetes have a significantly higher infectious risk compared to the general population. Furthermore, chronic hemodialysis generates additional chronic inflammation and a defective acquired immune response.

Multiple studies have reported the increased risk of lower respiratory tract infections such as pulmonary tuberculosis [2,3,4,5] and pneumonia [6,7,8,9], urinary tract infections [10,11], and skin and soft tissue infections [12,13,14,15] in people with diabetes. The outcome of infection treatment in patients who suffer from diabetes tends to be poor [10,15]. Because the immunological alterations caused directly by the diabetes are doubled by those secondary to hemodialysis in our group of patients (individuals on hemodialysis that also have diabetes mellitus), we will discuss these alterations simultaneously. Thus, we will present both the changes in the innate immunity and in the adaptive immunity (humoral and cellular immune responses) in this group of patients that are permanently under aggression, with the purpose of finding new therapeutic ways of improving the functionality of the immune system.

## 2. General Mechanisms of Endothelial Alterations in Patients with Type 2 Diabetes Mellitus

Unlike type 1 diabetes mellitus (T1DM), which is in the minority, type 2 diabetes mellitus (T2DM) makes up for approximately 90% of diabetes cases and this is why it is the subject of this study. T2DM is initially characterized by an increased peripheral insulin resistance, while later on it is represented by an insulin secretion deficiency in the β-pancreatic cells.

Insulin resistance consists of a decreased response (sensitivity) to insulin from the peripheral cells (especially hepatocytes, myocytes and adipose cells). In the first stages of diabetes, the β pancreatic cells still have functional reserves of insulin and they enter into a state of hyper-function as a result of the reduction in insulin peripheral sensitivity; thus, they are able to maintain normal glucose serum levels [16,17,18].

Over time, the β pancreatic cells’ function starts to decline and an insulin secretion deficiency develops. Accordingly, normoglycemia can no longer be maintained and hyperglycemia develops.

Patients with chronic insulin resistance (over 10 years old), which is associated with obesity, sedentarism and old age, require chronic insulin administration. This long-term insulin resistance is the most frequent cause of macro- and micro-vasculopathy, which primarily manifest at the level of the kidneys, peripheral nerves and retinal vessels [15]. The basis of this vasculopathy is the metabolic syndrome, which is actually represented by several different alterations that precede and accompany T2DM. Generally speaking, the aforementioned alterations refer to dyslipidemia and its pro-atherogenic effect and increases in blood pressure and in blood sugar levels and a prothrombotic status [19].

We have evidence that the metabolic syndrome is associated with low-grade chronic inflammation that may amplify the metabolic alterations through the actions of proinflammatory cytokines on myocytes and adipocytes, inducing an elevated resistance to insulin [20,21].

If the patients with diabetes also suffer from obesity, the adipocytes secrete an excessive amount of adipokines, in addition to the pre-existing ones. There are already comprehensive data about the effects of adiponectin, leptin, resistin and visfatin in diabetes mellitus [22,23,24]. 

Adiponectin and leptin are the two most abundant adipocytokines. Adiponectin is synthesized mainly by adipocytes. It suppresses macrophage functions and inflammatory processes and decreases insulin resistance. In contrast to adiponectin, serum levels of leptin are increased in people with obesity and leptin also has pro-inflammatory properties. Leptin links nutritional status with neuroendocrine and immune functions. Resistin, another pro-inflammatory adipocytokine, is involved in the regulation of insulin resistance and has many pro-inflammatory functions. Visfatin mimics insulin functions and thereby decreases insulin resistance [22]. Serum adiponectin levels are elevated in patients with diabetic retinopathy or neuropathy, and serum leptin levels are significantly higher in patients with neuropathy [25].

Another cause of endothelial disfunction in patients with diabetes is the activation of the alternative pathways of glucose metabolism. A higher than normal level of glucose can induce polyol, hexoamine and PKC (protein kinase C) pathways activation and increase basic glycolysis, as well as enhance AGEs (advanced glycation end products) synthesis [26].

A key factor involved in the development and progression of diabetic arteriopathy and neuropathy is increased glycosylation, a process in which glucose and other sugars interact with proteins. Glycosylated proteins become dysfunctional and are called advanced glycosylation end products (AGEs). High levels of AGEs have the ability to destroy cells or disrupt their functions. Two mechanisms are implicated: direct action and generation of an inflammatory reaction. These actions of AGEs are mediated through membrane cell receptors (RAGE) expressed on different cell types, including monocytes/macrophages, T lymphocytes, endothelial cells, smooth muscle cells, fibroblasts, mesangial cells and neurons. Increased vascular permeability, affecting several organs is secondary to the AGEs–RAGE interaction. After the ligation of RAGE, AGEs are endocytosed and activate the cells causing the production of inflammatory cytokines and growth factors, which in turn have been implicated in vascular pathology [27]. Hyperglycemia and tumor necrosis factor α (TNFα) stimulate the expression of RAGE [28].

One of the glycated proteins involved is hemoglobin, which has been used for years to monitor diabetes treatment. Hemoglobin glycosylation occurs at the level of the terminal amino groups (-NH2) in the α and β chains. Another glycated protein is spectrin, a major component of the erythrocyte membrane. The glycation process results in reduced red blood cell (RBC) deformability and an increased adherence to the endothelium [28].

Platelet membrane proteins can also be glycated. The increased fibrinogen binding and platelet aggregation observed in patients with diabetes can be linked to the glycation of adenosine diphosphate receptors [29].

The collagen and elastin from the vascular base membrane accumulate AGEs, resulting in secondary thickening of the basal membrane, which is a common feature of diabetic vasculopathy [29].

Diabetic retinopathy involves basal membrane thickening, a loss of pericytes, in-creased permeability and vascular dysfunction in the retinal capillaries. These alterations generate macular edema (due to the leakage of lipoproteins into the retinal layers) and progressive capillary closure through microthrombosis (ischemic retinopathy). The result is the secretion of vascular endothelial growth factor (VEGF), with secondary development of new vessels (proliferative retinopathy) [30].

Diabetic nephropathy (DN) is another progressive complication of diabetes, which typically and initially manifests as microalbuminuria. This phenomenon is induced by the thickening of the glomerular basal membranes and by glomerular hyperfiltration and leads to mesangial extracellular matrix expansion. The accumulation of glycated collagen in the glomerular extravascular matrix causes progressive capillary occlusion. The end results are an increase in urinary albumin excretion and the progression towards glomerular and tubular sclerosis and renal failure [30,31].

Another alternative metabolic pathway present in DM2 is the polyol pathway. Under normal cellular conditions, the aldose reductase (AR) reduces toxic aldehydes to inactive alcohols. In prolonged hyperglycemia, AR also reduces glucose to sorbitol. Sorbitol molecules are osmotically active molecules that generate hyperosmolarity in cells. The sorbitol thus obtained is subsequently oxidized to fructose. This reduction reaction consumes NADPH (reduced nicotinamide adenine dinucleotide phosphate), an essential cofactor implicated in reduced glutathione regeneration (which is a key mechanism of antioxidative cellular protection). The depletion of NADPH in the polyol pathway thus increases cell susceptibility to intracellular oxidative stress [31,32].

The excessive amounts of fructose obtained through sorbitol oxidation generate the protein fructosylation (Maillard reaction), which represents fructose binding to amino acids [33,34]. Initially, a small number of amino acids are fructosylated. This process is followed by the formation of reactive protein intermediary products and, later on, by that of cross-linking proteins. Fructosylation also generates additional AGEs, with decreased protein quality due to the loss of amino acid residues and decreased protein digestibility. The protein compounds generated through glycosylation and fructosylation can inhibit the uptake and metabolism of free amino acids and of zinc. Some advanced Maillard products have mutagenic and/or anticarcinogenic properties [35].

Fructosylation can also generate neo-epitopes. The alteration in the structure of human serum albumin in vitro makes it more potent as an antigen compared to its native form [35].

All of the aforementioned general mechanisms act on all tissues. The heart, brain and peripheral vasculature are affected by both diabetic macrovasculopathy and microvasculopathy; on the other hand, the retina and kidneys are mainly affected by microvasculopathy. Thus, diabetes mellitus does often also lead to renal insufficiency and the necessity of hemodialysis.

Moreover, a new concept was proposed to offer an explanation for all of these vascular alterations in diabetes: diabetic panvascular disease (DPD). It is defined as a clinical syndrome in which atherosclerosis is the connecting pathology between macrovascular and microvascular alterations in patients with diabetes [36].

## 3. Participation of Endothelium in Diabetic Inflammatory Status

The endothelium can be viewed as a semipermeable membrane with an essential role in maintaining tissular homeostasis (a basic, key concept in medicine, which refers to a tissue’s or organ’s ability to maintain a stable, balanced, physiological state). The term comes from Greek and relates to all the processes through which a tissue is able to correct any imbalances created by stimuli. The endothelium allows nutrients and leukocytes to pass from the blood to the tissues when needed, while simultaneously blocking the passage of microorganisms and certain toxic compounds. The endothelial cells also control vascular tone and coagulation, vascular cell growth and immune responses [15].

The most widely known and studied endothelium-produced mediator is the nitric oxide (NO). In order to produce it, the endothelial cells activate their specific synthase, NOS_3_ (eNOS). There are three synthases that have been discovered so far: NOS_1_ or the neuronal synthase, NOS_2_ or the inducible synthase and NOS_3_ or the endothelial synthase. NOS_1_ and NOS_3_ are physiological synthases, while NOS_2_ is specific to inflammatory reactions [15].

The NO produced by NOS_3_ diffuses into the vascular wall, reaching up to the smooth muscle cells, where it activates the guanylate cyclase (GC) that produces cyclic GMP (cGMP). In turn, the cGMP induces vascular relaxation [15,37].

Concomitantly, the NO inhibits platelet aggregation, smooth muscle cell proliferation and the transcription of vascular adhesion molecules, especially vascular cell adhesion molecules (VCAMs) and intercellular adhesion molecules (ICAMs) [37]. NO synthesis is stimulated by the hemodynamic sheer stress and acetylcholine and bradykinin agonists. Regardless of the signaling mechanism employed, the two agonists increase NOS_3_ expression.

There are other important vasodilators besides the NO, including EDHF (endothelium-derived hyperpolarizing factor) and prostacyclin (PGI_2_), which also acts as a thrombocyte inhibitor [15].

In order to maintain equilibrium, the endothelium also secretes endothelin-1 (ET-1), a strong vasoconstrictor, thromboxane A2 (TXA_2_), prostaglandin H2 (PGH_2_) and reactive oxygen species (ROS). Moreover, the endothelial cell expresses ACE (endothelial cell-bound angiotensin-converting enzyme), which catalyzes the transformation of angiotensin I (AG I) into angiotensin II (AG II) [15,37].

The healthy, intact endothelial cell does not allow leukocyte adhesion, while in inflammation (in the inflammatory site), the endothelium will express leukocyte adhesion molecules and will excrete proinflammatory cytokines (like TNFα), that amplify inflammation [38].

The endothelium also participates in the control of VEGF (vascular endothelial growth factor)-induced angiogenesis, which activates the mitogen-activated protein kinase (MAPK) cascade. When angiogenesis is not necessary, the endothelial cells oppose VEGF action by synthetizing antiangiogenic factors, like angiostatin and thrombospondin [15].

The aforementioned mechanisms characterize the normal vascular behavior. In pathological conditions, vasoconstriction, proinflammatory and prothrombotic mechanisms will be predominant, generating atherosclerosis. Thus, in T2DM, there appears to be a reduction in vasodilation capacity, but the mechanisms behind it are not fully elucidated, since there are more factors involved than just hyperglycemia [15,38].

**Figure 1:**
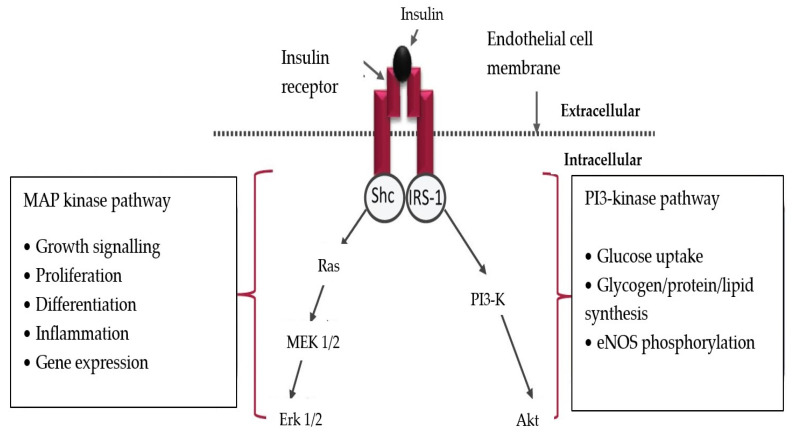
The effects of insulin on the endothelium. IRS-1: insulin substrate receptor-1; PI3-K: phosphatidylinositol 3-kinase; Akt: protein kinase B; MEK 1/2: mitogen-activated protein kinase; Erk 1/2: extracellular receptor kinase; eNOS: endothelial nitric oxide synthase (Reproduced from source: Roberts AC, Porter KE, 2013 [40]).

Physiologically, insulin triggers endothelial signaling through the PI-3K (phosphoinositide-3 kinase)/Akt pathway (Figure 1). The result is NO synthesis, through eNOS activation. In T2DM, because of the increased insulin resistance and the functional deficiency of the PI-3K/Akt pathway, the MAPK will be predominantly activated, which promotes atherosclerosis. As a result of the deficient signaling through the PI-3K/Akt pathway, NO is reduced—a fact initially proven on obese mice [39].

## 4. Proinflammatory Signaling in T2DM

In T2DM, due to the presence of a chronic inflammatory status, the endothelial cells adopt the pathological model of behavior (as described above), thus generating the vascular complications of diabetes. There are many factors acting upon the endothelial cells, including proinflammatory cytokines, chemokines, adhesion molecules and transcription factors [41].

A study performed on obese animal models has identified the adipose tissue as the main source of proinflammatory cytokines, especially TNFα. It binds to its receptors (TNFR) and induces NF-kB activation. It was already proven that high TNFα and IkB (inhibitor of nuclear factor kappa B) kinase levels mediate the appearance of insulin resistance [42].

Free fatty acids (FFAs), which have high concentrations in T2DM, also activate NF-kB and the RAGE (receptor for advanced glycation end-products) receptor. 

At this point in time, we can conclude that NF-kB activation and the entrance of this transcription factor into the nucleus determines the expression of the proinflammatory proteins genes and that the resulting inflammation is associated with insulin resistance.

Additionally, inflammation significantly decreases NO endothelial production, through the physiological PI3-K/Akt pathway and induces atherosclerotic lesion formation.

## 5. The Role of Vascular Inflammation-Associated Oxidative Stress in T2DM

**Figure 2:**
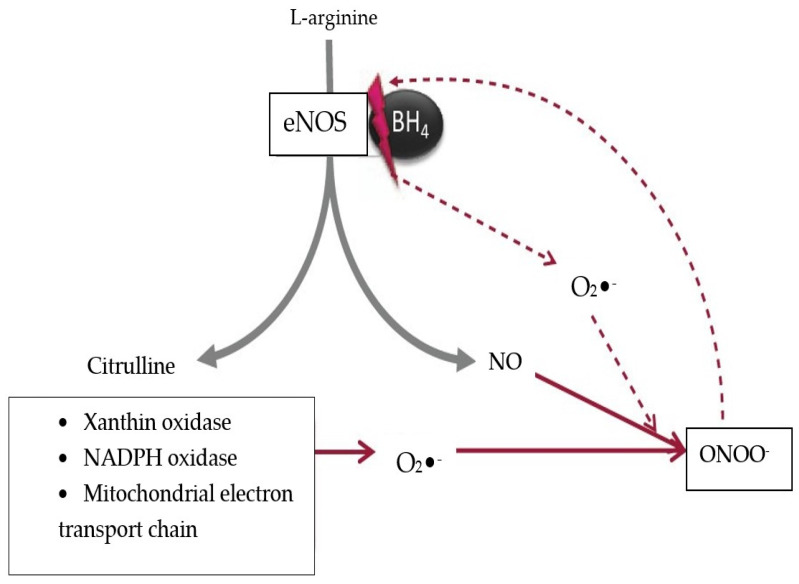
The production of peroxynitrite in the endothelial cells. In normal cells, the eNOS catalyzes NO synthesis from L-arginine. The factors inside the text box react with NO to produce ONOO^−^. The superoxide separates the eNOS from its cofactor BH_4_, increasing O_2_^·−^ and ONOO^−^. NO: nitric oxide; ONOO^−^: peroxynitrite; eNOS: endothelial nitric oxide synthase; O_2_^·−^: superoxide anion; BH_4_: tetrahydrobiopterin; grey arrows: physiological processes; red arrows: pathological processes (Reproduced from source: Roberts AC, Porter KE, 2013 [40]).

All inflammatory reactions are associated with the production of reactive oxygen species (ROS) by the leukocytes. The first ROS to be synthetized is the superoxide anion (O_2_**^·−^**). NO is also produced in the inflammatory site, in its radical form, through the activation of NOS_2_. The NO radical reacts with the superoxide anion and produces the peroxynitrite radical (ONOO^−^), a very strong oxidizing compound that is capable of acting upon many different molecules, including those of the physiological PI3-K/Akt pathway (Figure 2) [43].

The peroxynitrite can also block tetrahydrobiopterin (BH4), the cofactor of NOS_3_ (endothelial NOS). In this way, instead of physiological NO, it is the radical form which predominates in inflammation.

Considering all the facts mentioned above, the peroxynitrite radical can be perceived as an inhibitor of the physiological PI3-K/Akt pathway [40,44].

There are other sources of superoxide anions, besides eNOS synthesis, such as the xanthinoxidase, NADPH- oxidase (nicotinamide adenine dinucleotide phosphate oxidase) and mitochondria. In physiological conditions, the mitochondrial synthesis of ROS is negligible, but becomes significant in diabetes, when hyperglycemia induces an increase in proton concentration gradient inside the electron transportation chain [45].

Patients with T2DM have shown an intensification in mitochondrial fission and fragmentation, which increase ROS production [46].

## 6. The Molecular Mechanisms of Increasing Insulin Resistance Induced by Inflammation

After insulin binds to its transmembrane receptor, a signal is triggered that will initiate tyrosine phosphorylation in IRS-1 and IRS-2 adaptor proteins (insulin receptor substrates 1 and 2). These are attached to the cytosolic end of the receptor. The end result is the physiological effects of insulin.

**Figure 3:**
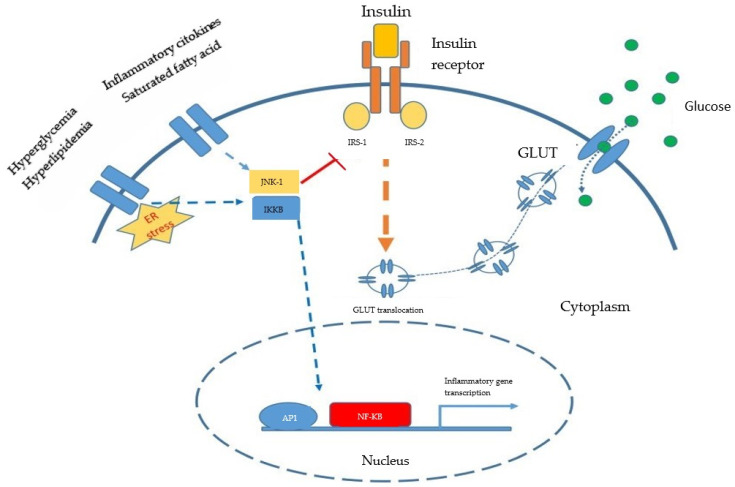
The molecular mechanism of insulin resistance due to inflammation (Reproduced from source: Odegaard JI, Chawla A, 2011 [48]).

When insulin attaches to its receptor on the external membrane, the cytoskeletal proteins are activated through phosphorylation. Thus, the glucose transportation vesicles, GLUT (GLUT4), are moved towards the membrane (Figure 3). The transporter remains embedded in the membrane and acts as a channel for glucose. The mere presence of insulin on the cellular membrane increases glucose transmembrane transportation tenfold (compared with insulin absence). This phenomenon was mainly observed in the adipose tissue, striated muscle cells and myocardial cells [47].

If the serine in IRS-1 and IRS-2 is phosphorylated instead of tyrosine, via IKKβ (inhibitor of nuclear factor kappa-B kinase subunit beta) and JNK1 kinases (c-Jun N-terminal kinases 1), insulin signaling is blocked, thus increasing insulin resistance. The same two kinases also activate gene transcription for multiple proteins involved in inflammation. The kinases are activated through increases in the concentrations of glucose, fatty acids, diacylglycerol (DAG), TNFα, IL-6, C reactive protein (CRP), ROS and hypoxia. After activation, IKKβ induces NF-kB translocation into the nucleus and gene transcription for proinflammatory proteins and also for the proteins involved in the immune responses. JNK activation through TNFα inhibits insulin induced signaling towards the IRS-1 adaptor protein [49].

In conclusion, this appears to be the mechanism through which inflammation is self-maintaining and insulin resistance is generated.

## 7. The Inhibition of Leukocyte Chemotaxis in T2DM

A study carried out on both human and animal diabetic subjects has shown a reduction in leukocyte infiltration in virus- or bacteria-infected tissues. Both CD45+ and CD8+ cytotoxic lymphocytes were affected. Moreover, a reduction in the expression of ICAM-1 (intercellular adhesion molecule 1) and CAM (cell adhesion molecules) E-selectin-type adhesion molecules was also observed. A decrease in inflammatory cytokines (IL-1β, TNFα) production was also reported in pulmonary tissue infected by K. Pneumoniae [50].

## 8. Alterations of Antigen Recognition in T2DM

There are many studies concerning the behavior of TLR signaling receptors on the neutrophils’ and monocytes’ membranes in patients with diabetes mellitus, precisely because these receptors have essential roles in both innate and acquired immunity. These are the only signals that find their way into the leukocyte genome and induce changes in the synthesis and release of proteins. Patients with diabetes with poorly controlled glycemia (and the resulting consequences) have been shown to experience a reduction in TLR receptors. In contrast, patients with good control over their blood sugar levels have shown normal, if not increased, TLRs [51].

## 9. Neutrophil Dysfunctions

Patients with diabetes have been proven to experience a decrease in degranulation and ROS (reactive oxygen species) synthesis and release. The first studies on neutrophil bactericidal capacity were performed on patients with diabetes and tuberculosis.

These neutrophil alterations were associated with an increase in resistin serum levels [52]. Initially, resistin was thought to be an adipokine (a hormone secreted by adipose cells). Later on, resistin was also discovered to have an important link to obesity, insulin resistance and diabetes. Furthermore, resistin is not only produced by adipose cells, but significant amounts also come from neutrophils, macrophages, splenic cells and bone marrow. Currently, resistin is known to be involved in atherosclerosis, cardiovascular diseases, chronic renal insufficiency (including hemodialysis), autoimmune diseases, inflammatory bowel diseases, malignancies, asthma. Also, an increasing number of studies attest to resistin’s role in chronic inflammation, endothelial disfunction, thrombosis, smooth muscle cell disfunctions and angiogenesis [52,53,54,55].

Neutrophils isolated from healthy individuals that were treated with a high-concentration glucose solution had a reduced synthesis capacity for superoxide anion (O_2_^•−^ and a decreased degranulation capacity [56,57].

**Figure 4:**
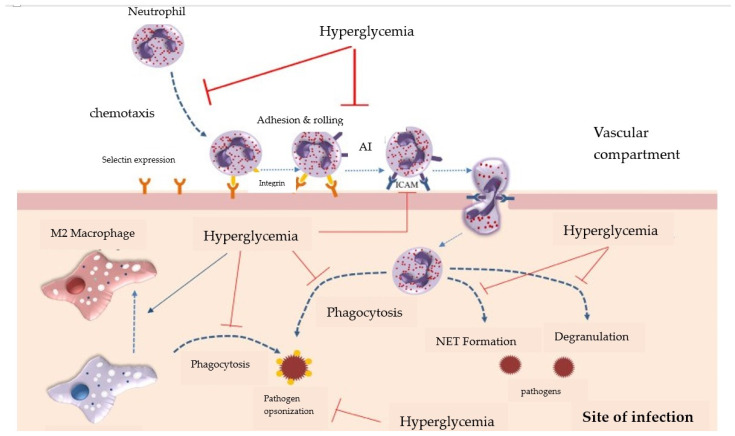
Impairment of immune response mechanisms during hyperglycemia (Reproduced from source: Ley K et al., 2007 [60]).

Several other studies suggest that hyperglycemia generates many dysfunctions in the neutrophils besides ROS production and degranulation alterations, including the inhibition of immunoglobulin opsonization of non-self structures and a reduction in NET (neutrophil extracellular traps = a network of extracellular strings of DNA that bind pathogenic microbes) formation and phagocytosis (Figure 4) [57,58,59].

## 10. Macrophage Disfunction

Physiologically, macrophages have receptors for the complement (CR) on their membranes and receptors for G immunoglobulins/IgG (FcγR), also known as endocytosis receptors. They bind to non-self structures opsonized with C3b or IgG. Chronic hyperglycemia is associated with defects in the endocytosis receptors, which generate phagocytosis alterations [61].

Another effect that hyperglycemia has on macrophages is their transformation to the M2 profile or alternatively activated macrophages. They have specific actions in the tissue regeneration and repair period at the end of inflammation and also present a reduced bactericidal capacity [62].

## 11. Natural Killer Lymphocyte Disfunction

Although they are a part of the same family as T and B lymphocytes, the Natural Killer (NK) cells/lymphocytes functionally pertain to innate immunity. They have destructive effects upon virally infected or malign cells and can also be found in the placenta. NK lymphocytes do not require epitope presentation by APCs (antigen presenting cells), they can directly induce the destruction of virally infected or malign targets. This functional aspect gave them the name of “natural killer” cells. NK lymphocytes communicate with macrophages and dendritic cells, stimulating them via IFNγ (interferon gamma) and TNFα (tumoral necrosis factor alpha). Patients with diabetes present with alterations to their NKG2D and NKp46 receptors, which generate defects in NK cell degranulation [63].

## 12. The Impact of Hemodialysis on the Immunity of Patients with Diabetes

More than 10% of the world’s population is suffering from chronic kidney disease (CKD). At the moment, 2.6 million people require hemodialysis and estimates show that this number will increase to 5.4 million in 2030 [64].

Although hemodialysis keeps the patients alive, it generates chronic systemic stress, via both hemodynamic and non-hemodynamic factors, resulting in an increased risk of cardiovascular, neoplastic and infectious diseases [65,66].

Patients on hemodialysis are also exposed to an almost constant activation of innate and adaptive immunity, resulting in chronic systemic inflammation [67]. This type of inflammation has been called “inflamm-aging” and it represents a persistent inflammatory status, which is low intensity and sterile and it is associated with immune system senescence [68].

Cellular senescence consists of permanent arrest of the cell cycle, the impossibility of proliferation, the expression of anti-proliferative markers and the shortening of telomeres. It is a proactive mechanism against malignity [69].

The accumulation of somatic senescent cells contributes to organ dysfunction and immune system alterations. Immune senescence is one of the causes of increased SASP (somatic cell senescence-associated secretory phenotype) production, because the immune cells present a decreased chemotaxis toward somatic senescent cells [70]. Senescent neutrophils and macrophages present a reduced phagocytosis capacity [71]. Moreover, the involution of the thymus is accompanied by a decreased ability to negatively select self-/auto-reactive T cells [72].

Many patients on hemodialysis acquired CKD as a result of complications of diabetes mellitus. In these individuals, we add to the pre-existing complications an additional systemic stress generated by the rapid hemodynamic alterations that occur during hemodialysis (loss of weight, fast ultrafiltration), the quick serum fluctuations of soluble compounds and electrolytes, the treatment programs and the mechanical interaction between the blood and the extra-corporeal circuit [73].

The roles of adipokines in diabetes mellitus have already been mentioned above. These adipocytokines also have important effects in patients with kidney disease. Leptin appears to be elevated in patients with ESRD, while increased levels of visfatin are linked to endothelial dysfunction, atherosclerosis and lipid dysregulation in patients with CKD. Resistin is associated with poor GFR [74].

### 12.1. Innate Immunity Alteration in Patients on Hemodialysis

#### 12.1.1. Complement System Alterations

During hemodialysis, the patient’s blood comes into contact with various acetylated compounds, carbohydrates and proteins adsorbed onto dialysis biomaterials. This phenomenon leads to C3-convertase activation, in all three complement pathways: the classic, alternative and lectin pathways [75]. This activation is proven in several ways: the increase in C3 concentration in the first 10–15 min of hemodialysis, followed by an increase in C5a and C5b levels and also an elevation up to 70% in serum MAC (membrane attack complex) levels and C3d/C3 ratio during one single hemodialysis session [76].

It is important to mention that these elevations in complement activation occur at the beginning of hemodialysis treatment and progressively decrease as the dialysis duration increases [77].

The first studies concerning serum complement behavior during hemodialysis have shown alternative pathway activation when cellulose-based dialysis membranes are used. Afterwards, classic and lectin pathway activation were also observed, generated by MBL (mannose binding lectin) and ficolin-2 or, respectively, properdin and/or C3b binding to the dialysis membrane [78,79].

Moreover, polysulfone dialysis membranes adsorb some complement inhibitors, like the H factor and clusterin and thus significantly reduce their serum concentrations and, implicitly, the complement inhibition capacity [80].

By utilizing filters with a medium separation capacity, hemodialysis induces a reduction in serum levels of many complement system components, including C4 and factor B. This decrease was not observed when polyamix membranes were used (highly selective membranes made out of a mixture of polyarylethersulfone, polyvinylpyrrolidone and polyamide) [81].

Applying various procedures to inhibit the complement system in patients undergoing hemodialysis has improved dialysis efficiency and long-term evolution, including the following:

- The administration of citrate, which inhibits platelet activation also blocks complement activity, through calcium chelation in the hemodialysis circuit [82];
- Administrating a C1 inhibitor has significantly reduced complement activation and IL-6 and TNFα proinflammatory cytokine release, as well as von Willebrand factor secretion [77];
- Blocking the complement system at C3 level through compstatin has led to increased dialysis membrane biocompatibility [83].

#### 12.1.2. Neutrophils and Monocyte–Macrophages Alterations

The systemic chronic inflammation generated by hemodialysis also presupposes serum neutrophil and monocyte activation. Once they cross from the vessels into the tissues, these monocytes become macrophages. Even so, at the end of a hemodialysis session utilizing polysulfone membranes, a reduction in the number of neutrophiles and a significant increase in monocyte and neutrophile apoptosis (but not of lymphocytes) were obtained. This explains the transitory leukopenia that appears during hemodialysis [84,85].

In vitro contact between blood cells and five different types of polysulfone dialysis membranes has showed important elevations in platelet adherence and neutrophile ROS production, along with an increase in fibrinogen adsorption. Only using low-fibrinogen-adsorption dialysis membranes can reduce the inflammation in the small caliber vessels and the oxidative stress that occurs during hemodialysis [86].

While neutrophils register a transitory drop in number during hemodialysis, monocytes present most of the functional and structural alterations. Monocytes can be divided into three types, according to their membrane markers: Mo1, Mo2 and Mo3. Mo1 monocytes have a classic membrane pattern—they have CD14 (lipopolysaccharides) receptor but do not express the CD16 (FcγIII) receptor for G immunoglobulins (IgG). Both Mo2 and Mo3 have the two receptors, CD14 and CD16 [81]. Mo2 type monocytes have a role in both innate (through transforming growth factor β (TGFβ) production) and acquired immunity (as APCs) [87]. 

Patients undergoing dialysis exhibit abnormally large numbers of Mo2 and Mo3 intermediary monocytes (CD14^++^/CD16^+^), that have proinflammatory and proatherogenic properties through their increased capacity to bind to endothelial cells (which will develop atherosclerosis and induce cardiovascular alterations) [88,89].

All types of analyzed monocytes have shown a significant reduction in membrane expression of CX3CR1 (CX3C motif chemokine receptor 1, also known as the fractalkine receptor or G-protein-coupled receptor 13) receptors in all patients on dialysis, which affects the monocytes’ capacity to adhere to the endothelium. This phenomenon has received in vitro confirmation, using monocytes that were incubated with uremic blood. The cells showcased immune alterations induced by hyperuricemia [89].

Moreover, the monocytes of patients undergoing dialysis treatment had an altered response to bacterial lipopolysaccharides stimulation, confirming the existence of an immunological disorder [90].

The type of dialysis membrane used also influences the number of monocytes. The most biocompatible membranes replace mostly Mo2 and Mo3 and less Mo1 [91]. The number of Mo3 monocytes reaches its lowest level 15–30 min after the start of the dialysis session and returns to normal at the end of the session, after 4–5 h [92].

#### 12.1.3. The Amplification of Chronic Inflammation in Patients with Diabetes Undergoing Hemodialysis

Patients with diabetes that are also undergoing hemodialysis treatment suffer from an amplification of the pre-existing chronic inflammation, generated by diabetes. This results in increased morbidity and mortality. The chronic inflammatory reaction is also influenced by the compatibility with the dialysis membrane, the patient’s age, associated infections, vascular access and existing comorbidities. There are several studies that attest to the increase in TNFα and IL-6 levels in adult patients on hemodialysis [93,94], as well as an elevation of IL-6 in children undergoing hemodialysis, compared with healthy children and those with stage 5 CKD [95].

Another inflammatory mediator that is typically altered in hemodialysis is endothelin 1 (ET-1). The chronic inflammation generated by both diabetes mellitus and hemodialysis leads to an increase in ET-1 levels, with a non-selective vasoconstricting effect (including on the coronaries). ET-1 is also a chemotactic factor for the leukocytes, as it induces the expression of leukocyte adhesion molecules on the endothelial membranes, especially those used for neutrophile adhesion [96]. 

The activation of leukocytes during inflammation also implies ROS production. Also, the mechanisms of ROS neutralization and suppression were impaired after only one session of hemodialysis [97]. This “pro-oxidative” status in hemodialysis patients can explain the oxidation of lipids and proteins, as well as the increased proinflammatory cytokine production, through NF-kB transcription factor activation. Another cause of oxidative stress is the presence of bacterial DNA inside the dialysis product, which can justify the high levels of C reactive protein (CRP) and IL-6 [98].

Another factor that justifies the existence of chronic inflammation in patients with diabetes mellitus undergoing hemodialysis is the appearance of very pronounced acid-base imbalances. Before the start of the hemodialysis session, all patients have low levels of serum bicarbonate, as a result of the reduction in renal proton elimination and bicarbonate reabsorption. Additionally, the insulin deficiency greatly reduces Krebs cycle functionality, forcing cells to commute to anaerobic glycolysis and produce large amounts of lactic acid. The resulting acidosis is further amplified by the lactic acid synthesis that occurs secondary to the hypoxia generated by the normochromic normocytic anemia in CKD. The acidosis generated through all the aforementioned mechanisms stimulates inflammation and, implicitly, ROS production [99]. Moreover, by attempting to partially correct the anemia with iron administration, we stimulated ROS production even further, through the amplification of the Fenton reaction [100].

Even the type of vascular access used influences the patient’s inflammatory status. Central venous catheters are associated with a high occurrence of associated infections and inflammation and a higher mortality rate, compared with arteriovenous fistulas [101].

### 12.2. Acquired Immunity Alterations in Patients Undergoing Hemodialysis

#### 12.2.1. T Lymphocyte Alterations

A study performed on hemodialysis patients has shown a reduction in the numbers of and a functional alteration to several types of T lymphocytes: naïve, LTH2 (which stimulate the humoral immune response) and LT reg/suppressor LTs [102]. On the other hand, we see an increase in highly differentiated memory LTs [103].

The reduction in T lymphocytes in this category of patients is caused by decreased bone marrow proliferation and thymus maturation, as well as an increase in apoptosis [104,105].

The frequent administration of recombinant erythropoietin (rhuEPO) for correcting anemia is one of the causes of LT CD4^+^ apoptosis [106].

Moreover, after one single hemodialysis session, the CD4^+^/CD8^+^ lymphocyte ratio was reduced, in addition to the LT CD4^+^ response capacity for mitogen stimulation [107].

The decrease in LT CD4^+^ numbers is attributed to the reduction in the number of cellular divisions and in the number of cells capable of division. Another cause is the increased amount of time necessary for the cells to enter the G1 phase of the cellular cycle [108]. The remaining LT CD4^+^ also show alterations: shortened telomers and a reduction in the membrane expression of antigenic receptors [108].

All these phenomena characterize stress-induced premature lymphocyte senescence, which has negative effects on organism defense, through both adaptive immunity and hemodialysis-induced chronic inflammation [109].

Because all acquired immune responses are associated with an innate inflammatory reaction (inflammation), we can also say that T lymphocytes do not only participate in adaptive immunity, but also induce innate immunity activation through the cytokines they produce. Thus, by amplifying the pre-existing systemic inflammation in the endothelial system, LTs also have a role in atheromatous plaque destabilization [110].

#### 12.2.2. B Lymphocyte Alterations

B lymphocytes have also shown a reduction in the number of naïve B cells and an increase in the highly differentiated LBs [111]. One possible explanation could be the elevation of the soluble CD40 serum level in hemodialysis patients, which antagonizes the normal interaction between the CD40 receptor on the membrane of LBs and its ligand CD40L on the membranes of LTs. Thus, the naïve LBs are prevented from exchanging information with LTh, so the humoral immune response is blocked and the naïve LBs have a severely reduced life expectancy [112].

## 13. Conclusions and Future Directions

Both diabetes mellitus and hemodialysis affect innate immunity (the inflammatory response) and acquired immunity (the humoral and cellular immune responses).

In this paper, we have outlined the mechanisms of endothelial alteration in diabetes and hemodialysis, along with the mechanisms of generating inflammation and signaling in inflammation, in the endothelium and tissues and also the mechanisms through which inflammation induces insulin resistance.

This study also discussed the alterations in leukocyte chemotaxis, antigen recognition and the dysfunctionalities in neutrophiles and macrophages, in order to create an outline of innate immunity alterations in patients with diabetes undergoing hemodialysis. Regarding acquired immunity, we have presented the behavioral alterations of T and B lymphocytes, induced by diabetes mellitus and chronic hemodialysis.

The purpose of this paper was to outline and compare the mechanisms that are the basis of the constant aggression towards self-components that affects patients with diabetes on hemodialysis, in order to find possible new therapeutic ways to improve the functionality of the immune system. As a new therapeutic approach, we would suggest the identification of blockers for the soluble receptors of proinflammatory cytokines (TNFα, IL-6), through which they produce proinflammatory and anti-regenerative effects [113,114]. These blockers would allow the aforementioned cytokines to act only upon fixed membrane receptors, thus stimulating their pro-regenerative capacity.

Insulin resistance is also increased through the effects of proinflammatory cytokines, especially adipocytokines (TNFα), at the endothelial level. Through their stimulation a proinflammatory status is maintained, which increases resistance to insulin and decreases NO production [42]. Therefore, we also propose the option of identifying blocking compounds for the endothelial receptors for TNFα and IkB kinase.

## Data Availability

We consent to data sharing in a publicly accessible repository.
